# Influences of human contact following milk-feeding on nonnutritive oral behavior and rest of individual and pair-housed dairy calves during weaning

**DOI:** 10.3168/jdsc.2022-0264

**Published:** 2022-10-08

**Authors:** S.B. Doyle, E.K. Miller-Cushon

**Affiliations:** Department of Animal Sciences, University of Florida, Gainesville 32611

## Abstract

Dairy calves are active around the time of milk-feeding and often perform nonnutritive oral behaviors, particularly during weaning. This study evaluated the interactive effects of social housing and human contact following feeding, including scratching to mimic brushing, on postfeeding nonnutritive oral behaviors and rest, during the beginning of the weaning period. We enrolled individually housed dairy heifer calves (n = 14) and pair-housed heifer calves (n = 14; 1 focal calf/pair). Human contact was provided in the form of scratching calves beneath the neck to mimic allogrooming. The human was present for 5 min, within the 15-min window following morning milk-feeding. Human contact and control days, where there was no change in the postfeeding routine, were randomized for each calf over the course of 4 consecutive days during weaning. Behavior was recorded continuously from video for 1 h following milk-feeding. Individually housed calves performed more pen-directed nonnutritive oral behavior than pair-housed calves, but provision of human contact reduced the duration of this behavior to a level that did not differ from pair-housed calves. Although human contact did not affect the duration of pen-directed nonnutritive oral behavior in pair-housed calves, cross-sucking was reduced in pair-housed calves when they received human contact. Human contact following milk-feeding reduced the total duration of all nonnutritive oral behavior (pen-directed, bedding-directed, cross-sucking, and human-directed) and increased rest with no effect of housing treatment or interaction between housing treatment and human contact. These results suggest that human contact influenced performance of nonnutritive oral behavior following milk-feeding, particularly reducing pen-directed sucking in individually housed calves, highlighting the role of restrictive environments in the expression of these behaviors in conventionally housed dairy calves.

## Highlights

• Calves reared in individual or pair pens were exposed to human contact, including scratching to mimic allogrooming, on certain days during the weaning period.
• Individually housed calves performed more pen-directed oral manipulation than pair-housed calves, but human contact following milk-feeding reduced the duration to a level resembling pair-housed calves.
• Human contact following milk-feeding reduced the total duration of nonnutritive oral behavior (including pen-directed, human-directed, and cross-sucking in pair-housed calves) and increased duration of lying for calves in both housing systems.

## Graphical Abstract

**Figure:**
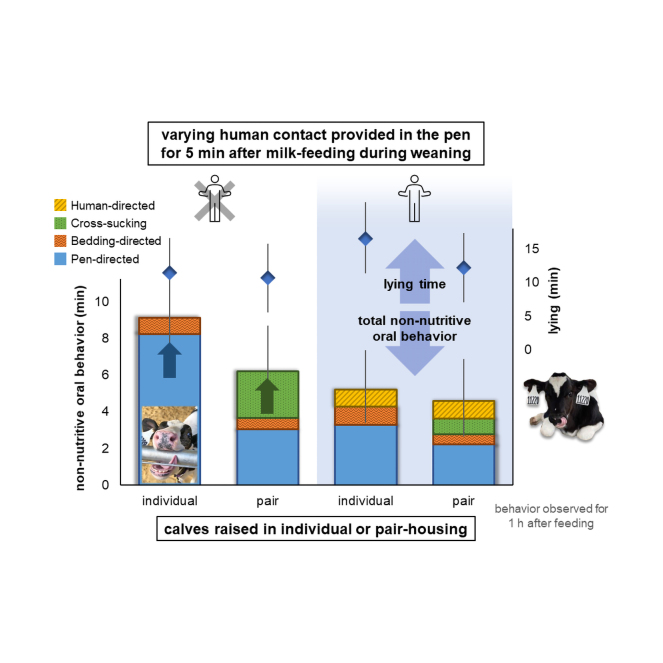
**Summary:** This study evaluated the interactive effects of social housing and human contact following feeding, including scratching to mimic brushing, on nonnutritive oral behaviors and rest of dairy calves during weaning. Individually housed calves performed more pen-directed nonnutritive oral behavior than pair-housed calves, but provision of human contact reduced the duration of this behavior to a level that did not differ from pair-housed calves. Human contact following milk-feeding reduced the total duration of all nonnutritive oral behavior (pen-directed, bedding-directed, cross-sucking, and human-directed) and increased rest with no effect of housing treatment or interaction between housing treatment and human contact. These results suggest that human contact influenced performance of nonnutritive oral behavior following milk-feeding, particularly reducing pen-directed sucking in individually housed calves, highlighting the role of restrictive environments in the expression of these behaviors in conventionally housed dairy calves.

Conventional rearing practices for dairy calves involving routine social isolation and restricted opportunities for natural feeding behavior have a range of detrimental effects on behavioral development. A common concern is the extensive performance of abnormal oral behavior in dairy calves, including pen-directed sucking and oral manipulation (Horvath et al., 2020; Downey et al., 2022), and cross-sucking in socially housed calves (Salter et al., 2021). Approaches to partially reduce these behaviors involve incorporating more varied and natural feeding behavior, including provision of suckling outlets such as rubber teats (de Passillé and Rushen, 1997; Horvath and Miller-Cushon, 2017; Salter et al., 2021), as well as providing access to forage (Haley et al., 1998; Horvath and Miller-Cushon, 2017, 2019; Downey et al., 2022). Despite these opportunities to address aspects of feeding behavior, environmental complexity for conventionally reared dairy calves remains quite limited and likely has implications for the development of abnormal repetitive behaviors.

Considering the human involvement in rearing calves on conventional dairy farms, human-animal relationships may be an important component of the dairy calf's early environment with implications for behavioral development. Gentle human handling has various positive effects on young calves, including reduced reactivity to human presence and improved ease of handling (Destrez et al., 2018; Lensink et al., 2001). Tactile human contact, including brushing, may be particularly beneficial. Westerath et al. (2014) found that heart rate was reduced following brushing, suggesting a reduction in arousal, and speculated that brushes were perceived positively, as calves leaned into the brushes and stretched their necks as brushing occurred. While effects of human contact and brushing on abnormal oral behaviors in calves have not been evaluated, Horvath et al. (2020) found that calves who were provided access to stationary brushes in their pen performed less nonnutritive oral behavior coinciding with milk-feeding and spent more time lying around the time of feeding, which may be interpreted as reduced arousal following feeding.

Since cattle are highly motivated to seek out grooming substrates, as demonstrated by McConnachie et al. (2018), and since calves often seek out human contact, as demonstrated by Lensink et al. (2000), it can be speculated that calves may find humans mimicking allogrooming rewarding. This type of human-animal interaction may be even more salient for calves who are housed individually without any physical contact with conspecifics. For example, previous findings suggest that individually housed calves initiate contact with humans more readily than pair-housed calves (Duve et al., 2012). However, there is a gap in knowledge concerning potential aspects of the human-animal relationship that may affect performance of abnormal oral behaviors in dairy calves. Therefore, the objective of this study was to evaluate the interactive effects of calf social housing and human contact on performance of nonnutritive oral behavior and rest following milk-feeding during the weaning period, as nonnutritive oral behaviors in dairy calves typically increase during weaning (e.g., cross-sucking; Salter et al., 2021). We hypothesized that provision of human contact, including scratching, shortly after milk-feeding would reduce the performance of nonnutritive oral behavior and increase rest following milk-feeding, given previous evidence of similar effects of brush access (Horvath et al., 2020). We additionally hypothesized that calves housed individually would seek out more contact with humans and would display more nonnutritive oral behavior compared with calves housed in pairs.

Holstein heifer calves (n = 28) were enrolled at the University of Florida Dairy Unit (Hague, FL) within 24 h of age. Calves were managed according to standard operating procedures for this facility and all study procedures were approved by the University of Florida Institutional Animal Care and Use Committee (#201910617). Calves received 4 L of quality-controlled colostrum by bottle within 2 h of birth (by bottle or, if calf would not suckle, by esophageal feeder) and were identified with radiofrequency identification ear tags. Calves were randomly assigned to either individual (**IH**; n = 14 calves) or pair housing (**PH**; n = 14 pairs, 28 PH calves total; one focal calf per pair enrolled) at birth, for the first 7 wk of life. Sample size was decided based on previous work examining effects of brush provision on duration of pen-directed nonnutritive oral behavior in individually housed calves (Horvath et al., 2020).

Pens were constructed of rigid wire mesh (10 × 20 cm grid openings) with dimensions of 0.9 × 1.8 m (width × depth) for individual pens and double-sized pens (1.8 m × 1.8 m; width × depth) for PH calves. Pens were placed within visual but not physical contact. Individual and paired pens were interspersed. Pens were sand-bedded and located within an open-sided barn where they were protected from downward rain and sun [average daily high temperature was 23.9 ± 5.2°C (mean ± SD) and daily low was 6.8 ± 6.0°C; historic weather data retrieved from www.wunderground.com]. All calves were provided with 8 L of milk replacer (28% CP and 20% fat; Suwannee Valley Feeds) in 2 meals/d via teat bucket at 0630 and 1430 h. Calf starter (Ampli-Calf Starter, Purina Animal Nutrition LLC) and water were provided to calves ad libitum. Calves did not receive forage during this experiment. During wk 4 of life, calves were disbudded by hot iron with a local cornual nerve block and nonsteroidal anti-inflammatory for analgesia. All human contact was standardized across treatments, and limited to feed delivery and routine health examinations, outside of human contact interventions described below.

Calves began weaning at 6 wk of age (42 ± 1.8 d of age; mean ± SD). Milk allotment was gradually decreased over the course of 10 d. For the first 4 d of weaning, calves received 6 L/d of milk rather than 8 L/d, divided in 2 meals/d. For the following 4 d, this was reduced to 4 L/d of milk provided in 2 meals/d. For the final 2 d before calves were fully weaned, they received 2 L/d in 2 meals/d.

For the first 4 d of weaning in wk 6, when milk allotment was reduced to 6 L/d of milk, calves were exposed to repeated periods of human contact, immediately following morning milk-feeding at approximately 0630 h, interspersed with control days with no additional human contact beyond standard management. Over the 4-d study period, the order of human contact and control days (4 possible options for order) were randomized for each individual calf using a random number generator. Over these 4 testing days, each calf experienced 2 d of a control treatment, where there was no change in their daily schedule, and 2 d of an experimental treatment involving human presence and interaction for 5 consecutive minutes within a 15-min window following morning feeding. A single human (to whom the calves were previously exposed during routine feeding) would briefly stand at the side of the pen and approach calmly before entering the calf pen. The 5-min human contact period would begin as soon as the human entered the pen. The human initiated contact with the calf upon entry to the pen. Calves were scratched beneath the neck and permitted to suck on the fingers or clothes of the human. If the calf did not contact the human or moved away, the human would wait for the calf to cease movement and then attempt to reinitiate contact. The human exited the pen after the 5-min period had elapsed, regardless of the duration of physical contact with the calf.

Since human contact was provided after milk meals, most calves were not lying down during treatment. However, if calves were already lying down, the human would simply crouch down to scratch beneath the neck of the calf rather to avoid disrupting them. When providing PH calves with human contact following milk-feeding, interaction was only initiated with the focal calf. If the nonfocal calf contacted the human, no attention was given to that calf unless they were disrupting contact with the focal calf, in which case the human would use a hand to gently block them. Contact was provided primarily by one human. If the number of calves assigned to receive human contact on a given day was such that a single human could not complete data collection within the 15-min postfeeding window specified, an additional human (with the same degree of familiarity and role in routine husbandry) would assist. The approach to calf contact and scratching (intensity and location on the neck) was consistent between humans and established during training before the study.

Neck scratching was chosen as the form of contact initiated by the human, based on evidence that calves utilize grooming substrates (such as brushes) with peaks in usage following feeding (Horvath et al., 2020). In the present study, scratching was implemented directly by the human using their hand, as opposed to holding a brush, to avoid potential avoidance or increased attention directed toward a novel brush. The 15-min window of intervention was selected to coincide with periods of increased sucking motivation and stimulus-seeking following milk-feeding. Motivation to engage in nonnutritive sucking behaviors appears to last approximately 10 min following a milk meal (de Passillé and Rushen, 1997; Haley et al., 1998), and previous work from our group found that postfeeding peaks in nonnutritive oral behavior were reduced through brush provision during this time window (Horvath et al., 2020).

Data were collected for 1 h, beginning at the start of the human intervention (or corresponding time point for control days), via video recorded from overhead digital video cameras (Axis M2026-LE Network Camera; Axis Communications). Observations began when a human entered the pen and began treatment or at the corresponding time point on control days, when milk-feeding concluded and the teats from the teat buckets used for milk-feeding were moved out of the calves' reach. Behavior was recorded continuously from video using Behavioral Observation Research Interactive Software (BORIS; Friard and Gamba, 2016), according to the ethogram described in Table 1. We recorded pen-directed nonnutritive oral behavior and bedding-directed nonnutritive oral behavior as separate behaviors, based on previous observations of calves in this housing system (Horvath and Miller-Cushon, 2017), which suggested that these behaviors were distinct in appearance. Pen-directed nonnutritive oral behaviors were previously observed most often and anecdotally involved tongue movements associated with feeding, resembling tongue rolling with the mouth placed over a pen bar (see here for a video example of this behavior: https://doi.org/10.6084/m9.figshare.20301480.v1; Miller-Cushon, 2022), whereas bedding-directed nonnutritive oral behavior involved licking, nibbling, and possible ingestion of bedding material. We had no specific hypothesis regarding how these different nonnutritive oral behaviors may respond to treatment due to limited understanding of differentiated causation. Tongue rolling was not observed, as it could not be reliably scored from video, but reports from live observation of preweaning dairy calves have suggested it occurs rarely (Downey et al., 2022). We additionally recorded self-grooming behavior, given previous evidence that it was increased when calves had access to a brush (Horvath and Miller-Cushon, 2019), to assess whether it was affected by scratching performed by a human.

**Table 1:** Ethogram describing behaviors of interest during both control and human intervention observations

Behavior	Description
Cross-sucking	Calf is using tongue or mouth to orally manipulate (chew, lick, or suck) the body part (excluding general licking of the coat, directed toward side or neck) or ear tags of another calf; described for pair-housed calves
Bedding-directed nonnutritive oral behavior	Calf mouth is in contact with the sand bedding from the ground; mouth is open, and calf may be licking or consuming sand
Pen-directed nonnutritive oral behavior	Calf mouth is open, and calf is using tongue or mouth to chew, suck, lick, or otherwise contact pen fixtures in any way with mouth movements visible
Human contact	Any physical contact between calf and human (initiated by calf or by human); recorded for the duration of human intervention (5 min)
Human-directed nonnutritive oral behavior	Calf is using tongue or mouth to lick, chew, or suck on person's clothing or body (defined as a subset of human contact); recorded for the duration of human intervention (5 min)
Lying down	Calf belly is touching the ground with front legs tucked beside or underneath the body and hind legs tucked beneath the body or splayed out beside the calf
Self-grooming	Calf uses mouth or tongue toward any part of their own body, or is scratching self with foot

These behaviors were recorded by a single observer with intraobserver reliability ≥93% (Cohen's kappa calculated in BORIS, based on viewing of one calf's complete video file). If the calf did not lie down, the maximum value of 60 min was assigned for latency to lie down. We additionally calculated total duration of nonnutritive oral behavior (sum of pen-directed, bedding-directed, human-directed, and cross-sucking).

Behavioral data were averaged for each calf across the 2 control days and 2 human contact days during the study period. Due to failed video recording, one day of human contact data was missing for 2 PH calves, and one day of control data was missing for 1 PH and 1 IH calf. Data were analyzed in a general linear mixed model (Proc MIXED in SAS v. 9.4; SAS Institute Inc.) with fixed effects of housing treatment (IH or PH), contact (human contact or control), and their interaction, and calf as a random effect. In the case of significant interactions between housing treatment and human contact, pairwise comparisons were examined using the Tukey-Kramer adjustment. Model residuals were screened for normality and durations of nonnutritive oral behaviors and lying down were square-root transformed to meet model assumptions of normality. All values reported are least squares means, with back-transformed means and 95% confidence intervals reported for data that were transformed to meet model assumptions. Significance was declared at *P* < 0.05, and trends were reported if 0.05 ≤ *P* ≤ 0.10.

When the human was present in the pen, there was contact between calf and human (including both calf-initiated contact and human touching the calf) for most of the test time (IH: 300 ± 0.1 s, mean ± SD; PH: 280.0 ± 61.1 s, mean ± SD). The duration of human-directed nonnutritive oral behavior did not differ between calves on different housing treatments (Table 2). Calves housed individually performed more pen-directed sucking when they did not receive human contact following feeding, but human contact reduced the duration of pen-directed nonnutritive oral behaviors to a level that did not differ from PH calves (Table 2). In contrast, human contact had no effect on duration of pen-directed nonnutritive oral behavior in PH calves. Bedding-directed nonnutritive oral behavior occurred less than pen-directed sucking and was not affected by housing treatment or human contact. The duration of cross-sucking in PH calves was reduced when they received human contact (Table 2).

**Table 2:** Effects of human contact^1^ (C) on behavior of dairy calves assigned to different housing treatments (T), either individual pens (IH; n = 14) or paired pens (PH; n = 14, with 1 focal calf/pair) during a 1-h period following milk-feeding during weaning

Item	No contact		Human contact		SE	*P*-value		
	IH	PH	IH	PH		T	C	T × C
Nonnutritive oral behavior^2^ (s)								
Human-directed^3^	—	—	56.0	57.9	—	0.88	—	—
			(39.4, 75.4)	(41.0, 77.6)				
Pen-directed	493.9^a^	183.4^b^	197.1^b^	133.0^b^	—	0.0028	0.0003	0.017
	(368.2, 638.1)	(110.4, 274.8)	(121.1, 291.5)	(72.2, 212.3)				
Bedding-directed	54.5	37.7	60.3	32.8	—	0.21	0.99	0.70
	(25.4, 94.5)	(14.4, 71.9)	(29.4, 102.2)	(11.5, 65.1)				
Cross-sucking	—	150.8	—	52.3	—	—	0.023	—
		(85.5, 234.7)		(17.6, 105.4)				
Total nonnutritive oral behavior^4^	575.8	441.7	353.7	342.7	—	0.39	0.0036	0.27
	(433.7, 738.0)	(318.5, 585.0)	(244.5, 482.9)	(235.4, 470.2)				
Self-grooming (s)	157.0	100.3	98.2	106.7	21.1	0.42	0.27	0.24
Lying down (min)	11.5	10.7	16.5	12.2	2.5	0.43	0.040	0.26
Latency to lie down (min)	34.7	38.2	32.6	40.9	4.0	0.27	0.90	0.32

a,b Means with different superscripts differ (*P*< 0.05).

1 Human contact, including neck scratching, was provided during a 5-min period within 15 min following the morning milk-feeding. Over a 4-d study period, calves received 2 d of human contact and 2 d where no contact was provided, in a random order.

2 Variables were square-root transformed to meet model assumptions of normality. Back-transformed means and 95% confidence intervals are reported.

3 Human-directed behavior was observed during the 5-min window within which calves received human contact.

4 Total nonnutritive oral behavior was calculated as the sum of human-directed (on days where human contact was provided), pen-directed, bedding-directed, and cross-sucking (for pair-housed calves). Note that because durations of nonnutritive oral behavior (both individual and total) were square-root transformed for analysis, this reported back-transformed mean value for “total nonnutritive oral behavior” is greater than the sum of the back-transformed means for each individual behavior.

There was an overall effect of human contact in reducing the total duration of nonnutritive oral behavior, with no effect of housing treatment (Table 2; Figure 1). Figure 1 illustrates these results, suggesting that the effect of human contact in reducing nonnutritive oral behavior was largely driven by the reduction in pen-directed sucking in IH calves, whereas the reduction in cross-sucking in PH calves was partly due to redirected behavior toward human-directed nonnutritive oral behavior.

**Figure 1:**
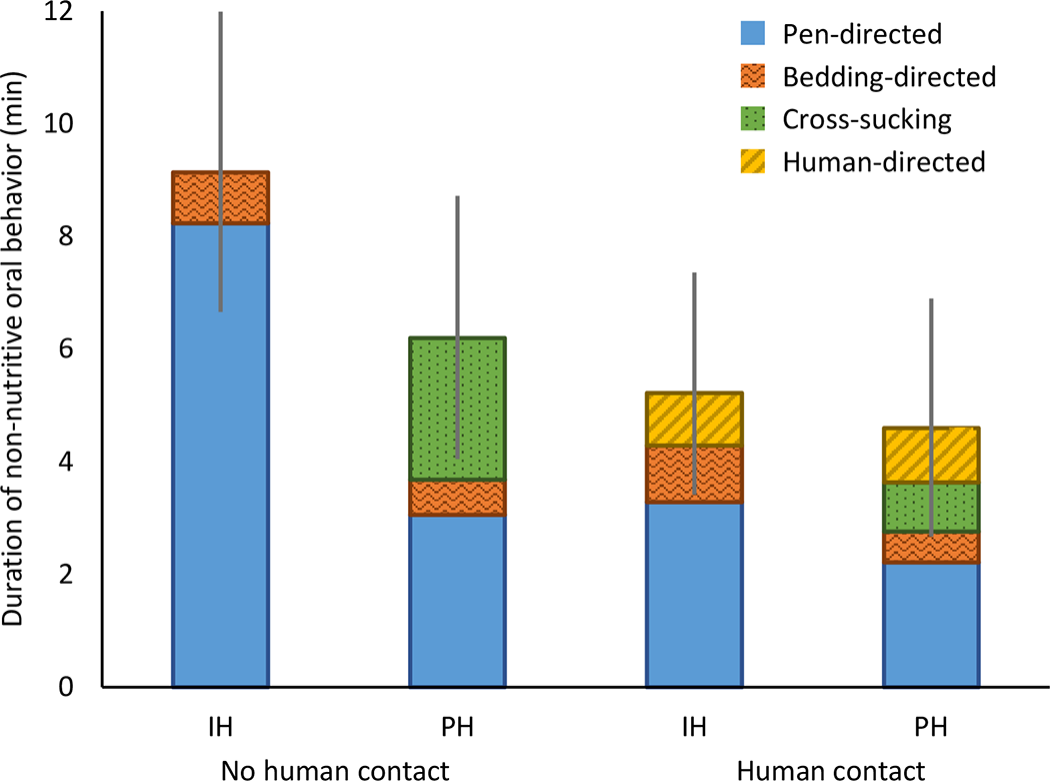
Stacked bar graph illustrating the effects of human contact on the durations of nonnutritive oral behaviors in individual (IH) and pair-housed (PH) dairy calves, observed for 15 min after milk feeding during weaning. Error bars depict the 95% CI for the cumulative duration of these behaviors (“total nonnutritive oral behavior” as presented in Table 2). Results of the statistical analysis of these individual measures are presented in Table 2.

Self-grooming was not affected by housing treatment or human contact. Calves spent more time lying down in the hour following feeding when they received human contact, with no effect of housing treatment. However, latency to lie down after feeding was not affected.

In support of our hypothesis, we observed that human contact reduced nonnutritive oral behaviors, particularly in IH calves, and caused an increase in lying time in the hour following feeding. Pen-directed oral manipulation, usually taking the form of tongue movement with the mouth over a pen bar, was the most prevalent form of nonnutritive oral behavior and was reduced both by social housing and human contact. Previously, this behavior has been observed frequently in calves in the same housing system (around an hour per 12-h observation period; Horvath et al., 2020), with peaks around milk-feeding but some performance throughout the entire day. In contrast, bedding-directed nonnutritive oral behavior (typically licking or ingesting bedding material) occurred to a lesser extent and was not affected by treatment, possibly suggesting that it is differently motivated from pen-directed oral manipulation. We found no effect of housing or human contact on self-grooming during our window of observation following feeding. Previously, we found that self-grooming was not affected by provision of a stationary brush (Horvath et al., 2020), although self-grooming was stimulated by access to a rotating brush (Horvath and Miller-Cushon, 2019).

Nonnutritive oral behaviors in dairy calves occur largely surrounding meals (de Passillé and Rushen, 1997; Haley et al., 1998) and our findings suggest that these behaviors are reduced through some aspect of human interaction. In general, calves are active and seeking stimulation following milk-feeding and also exhibit increased engagement with enrichment items, including brush use (Zobel et al., 2017; Horvath et al., 2020).

It is unclear whether effects of human contact in reducing nonnutritive oral behavior were due to some aspect of human presence or specifically the tactile experience of scratching, which was intended to mimic brushing. Previously, we found that provision of a brush reduced pen-directed sucking and increased rest surrounding milk-feeding times in calves (Horvath et al., 2020) and tongue rolling was also reduced in steers provided with brushes (Park et al., 2020). Although we did not observe differences in latency to lie down following feeding in the present study, increased lying time and reduced nonnutritive oral behaviors in the hour following feeding may suggest that some aspect of human contact reduced arousal. Westerath et al. (2014) found that dairy calf heart rates decreased following a period of brushing from humans. Apart from the physical sensation of brushing, human contact may also influence responses around feeding. Veal calves who were provided with positive human contact following milk meals displayed fewer avoidance behaviors, were easier to transport, and had reduced stress as indicated by decreased heart rate (Lensink et al., 2000). It is also possible that our observed effects may depend on the novelty of the human contact provided, as this was a short-term study focused specifically on the weaning period. Future work might explore effects of more recurring management strategies involving differing levels of human presence surrounding milk-feeding. While routine, prolonged human interaction is unlikely to be feasible on most dairies, these results suggest that even brief human contact surrounding milk-feedings may be beneficial in reducing nonnutritive oral behavior in calves.

Human contact was particularly influential in reducing nonnutritive oral behavior in IH calves compared with PH calves, and without human contact provided, IH calves performed more pen-directed sucking than PH calves. Evidence in other ruminant species also suggests that social isolation may increase nonnutritive oral behaviors (e.g., fence licking in giraffes; Bashaw et al., 2001). It is important to note that nonnutritive oral behaviors in milk-fed calves were not eliminated by either human contact or social housing in this experiment. In other studies, nonnutritive oral behaviors were only reduced but not eliminated by feeding management strategies (such as teat feeding and hay provision; Horvath and Miller-Cushon, 2017; Downey et al., 2022) or other environmental features (e.g., brush access; Horvath et al., 2020). These findings suggest a need for more complex or naturalistic feeding and social housing strategies for dairy calves to prevent abnormal oral behaviors and improve welfare.

Contrary to our hypothesis, we found no effects of housing treatment on human-directed behavior during the periods of human contact. Physical contact between calf and human was nearly constant in most cases, as the human initiated contact to scratch the calf, and the duration of human-directed nonnutritive oral behavior initiated by the calf did not differ between housing treatments. Previous findings suggest that calves prefer human contact more when housed individually (Duve et al., 2012; Lensink et al., 2000). In the present study, it is likely that the short period of observation and timing following feeding, selected as a time point where calves were engaged in more oral behavior, was not sufficient to reveal potential baseline differences in human-directed behavior.

In summary, we examined effects of human contact on nonnutritive oral behaviors of individual and pair-housed dairy calves during the weaning period. We found a decrease in pen-directed nonnutritive oral behaviors for calves housed individually as well as a lesser decrease in cross-sucking for pair-housed calves following human intervention. Human contact reduced the total duration of nonnutritive oral behaviors and increased rest following milk-feeding, across both housing treatments. Although the mechanism of these effects is unclear, whether related to an increase in environmental stimulation, a decrease in stress, or tactile stimulation, it could be suggested that providing positive human contact may be a form of enrichment for dairy calves. More generally, these results support a role of restrictive housing environments in the expression of abnormal oral behaviors, highlighting a need to consider more complex or naturalistic approaches to rearing dairy calves.
